# Editorial: Neuroinflammation and behavior

**DOI:** 10.3389/fnins.2015.00201

**Published:** 2015-06-02

**Authors:** Luba Sominsky, Adam K. Walker, Deborah M. Hodgson

**Affiliations:** ^1^School of Health Sciences, Health Innovations Research Institute, RMIT UniversityMelbourne, VIC, Australia; ^2^Laboratory of Neuroimmunology, Division of Internal Medicine, Department of Symptom Research, The University of Texas MD Anderson Cancer CenterHouston, TX, USA; ^3^Neuroendocrine Regulation of Cancer Laboratory, Monash Institute of Pharmaceutical Sciences, Monash UniversityMelbourne, VIC, Australia; ^4^Laboratory of Neuroimmunology, Faculty of Science and IT, School of Psychology, The University of NewcastleNewcastle, NSW, Australia

**Keywords:** neuroinflammation, neuroimmune, neuroendocrine, proinflammatory, mood disorders, microglia, perinatal programming, aging

Neuroimmune regulation plays a major role in many facets of human health. In the last 30 years our understanding of this intricate relationship between the brain and immune system has progressed immensely. Pioneering work in the late 1980s and early 1990s began to establish an understanding of the bi-directional communication between these previously considered distinct systems, and its implications in the regulation of mood and cognition (Dantzer and Kelley, 1989; Kelley and Dantzer, 1990; Bluthe et al., 1991). Since then, significant advances in the field of neuroimmunology have been made, and we now know considerably more about the mechanisms responsible for neuroimmune regulation of health and behavior. In this Frontiers research topic *Neuroinflammation and Behavior*, 10 groups of researchers contributed their expertise to discuss the recent knowledge in the field of neuroimmunology, focusing on the neuroinflammatory mechanisms in affective disorders, early life programming of neuroimmune function, as well as neuroimmune interactions in the aging brain and its associated pathologies.

Anisman and colleagues begin this topic with a review on the role of proinflammatory cytokines in depressive disorders, and the ability of social experiences to cause, exacerbate or mitigate cytokine imbalance and mood disorders. The authors provide evidence for a particular contribution of individual factors, such as sex, age, genetic, and other differences, to the effectiveness of social support in alleviating the symptoms of depression and neuroinflammatory signaling (Audet et al., 2014). Hutchinson and colleagues expand on this discussion by exploring the specific contribution of the innate immune pattern recognition receptor Toll-like receptor 4 (TLR4) in the pathophysiology of depression. The authors suggest that TLR4-mediated mechanisms could underpin the neural, immune, and neuroendocrine alterations seen in patients with major depressive disorder (Liu et al., 2014). Wohleb et al. propose a novel model of neuroimmune interactions that underlie psychosocial stress-induced immune and behavioral changes. In response to stress, simultaneous activation of peripheral monocytes and microglia induces peripheral and central inflammation. Neuroendocrine and sympathetic stress responses then promote monocyte trafficking to the brain and exacerbate microglial activation. This in turn contributes to the behavioral phenotype, such as persistent anxiety-like behavior (Wohleb et al., 2014). Baune and colleagues discuss the role of inflammasomes in neuroinflammation. Inflammasomes, by promoting the processing and activation of proinflammatory cytokines, play an instrumental role in the pathophysiology of neuroinflammation in the aging brain, as well as in the etiologies of several neurological diseases. Inflammasome-driven inflammation is also involved in the development of metabolic disorders that are known to be associated with mental illness (Singhal et al., 2014). This review highlights the therapeutic potential of targeting inflammasome-related pathways to combat neuroinflammatory diseases.

Several articles highlight the importance of early life events in the development of the neuroimmune circuitries. Campbell et al. investigate the role of the early microbial environment in the development of stress and nociceptive circuitries. The authors demonstrate that neonatal exposure to an immune challenge suppresses behavioral responses to nociceptive stimuli in adulthood. This is associated with increased activation of orexin neurons, implicated in stress and pain processing, in hypothalamic subregions (Campbell et al., 2015). These findings may have important implications for targeting the orexin system for treatment of chronic pain conditions, as well as highlighting the critical role of the early life environment in determining later health outcomes. The latter is further demonstrated by Spencer and colleagues. These authors examine the long-term effects of early nutritional environment on neuroinflammatory profile. Neonatal overfeeding induces basal microgliosis and increased neuronal activation in response to an immune challenge in the paraventricular hypothalamus. Interestingly, this early life intervention reduces central responses to the inflammatory effects of adult short-term high-fat diet. Whether these findings represent a suboptimal ability of neonatally overfed animals to counter the adversity of high-fat diet or whether this is an adaptive response, remains to be established (Cai et al., 2014). Jasoni et al. elaborate on the impact of perinatal immune-brain interaction by looking at maternal obesity-induced inflammation and predisposition of offspring to neurobehavioral and metabolic diseases. This review points to the common link between metabolic imbalance and neuroimmune activity and to the ability of the maternal immune milieu to alter fetal brain development and function long-term (Jasoni et al., 2014). The neuroinflammatory effects of obesity and other metabolic disorders persist throughout life, inducing cognitive dysfunction and other neuropathologies. Jenkins and colleagues review preclinical and clinical evidence concerning obesity and cognitive decline, focusing on the potential inflammatory mechanisms underpinning this neuropathology in obesity. The authors conclude that exercise may be the most efficient strategy to alleviate obesity-related cognitive impairment (Nguyen et al., 2014).

Fuggle et al. review the evidence for the impact of neuroimmune interactions in rheumatoid arthritis (RA) on the pathophysiology of this autoimmune disease. RA patients suffer from greater susceptibility to infections, further exacerbating joint pain and swelling. Current biologic therapies target the suppression of major inflammatory pathways involved in joint inflammation, improving disease control and quality of life. This, however, may result in even a greater susceptibility to infections, stimulating disease flares (Fuggle et al., 2014). This article highlights the importance of further research into therapeutic interventions for RA, sufficient to maintain disease remission, while preventing the degree of immunosuppression that may increase the risk of infections and trigger RA flares.

Finally, Walker and colleagues review the evidence that neuroinflammation plays a role in the neurobehavioral toxicities of chemotherapy. They focus on the common symptoms, such as cognitive decline, neuropathy and fatigue. While neuroinflammation has been thought to be the major mediator of these symptoms, the authors propose additional mechanisms may be involved, such as damage associated molecular patterns (DAMPS) and mitochondrial dysfunction (Vichaya et al., 2015). This article highlights the exciting potential for novel interventions to alleviate chemotherapy-induced neurotoxicities, significantly improving quality of life of many cancer patients and survivors.

In summary, the articles presented in this research topic provide a valuable insight into the importance of neuroimmune regulation in health and disease, encompassing a wide scope of conditions and presenting evidence for novel and intriguing mechanisms that underlie the reciprocal nature of brain-immune interactions.

## Conflict of interest statement

The authors declare that the research was conducted in the absence of any commercial or financial relationships that could be construed as a potential conflict of interest.
